# Biologically active substances-enriched diet regulates gonadotrope cell activation pathway in liver of adult and old rats

**DOI:** 10.1007/s12263-014-0427-1

**Published:** 2014-08-26

**Authors:** Hanna Oszkiel, Jacek Wilczak, Michał Jank

**Affiliations:** Department of Physiological Sciences, Faculty of Veterinary Medicine, Warsaw University of Life Sciences, Nowoursynowska 159 Str., 02-776 Warsaw, Poland

**Keywords:** Rat, Liver, Biologically active substances, Genes expression, GnRH

## Abstract

According to the Hippocrates’ theorem “Let food be your medicine and medicine be your food”, dietary interventions may induce changes in the metabolic and inflammatory state by modulating the expression of important genes involved in the chronic disorders. The aim of the present study was to evaluate the influence of long-term (14 months) use of biologically active substances-enriched diet (BASE-diet) on transcriptomic profile of rats’ liver. The experiment was conducted on 36 Sprague–Dawley rats divided into two experimental groups (fed with control or BASE-diet, both *n* = 18). Control diet was a semi-synthetic diet formulated according to the nutritional requirements for laboratory animals. The BASE-diet was enriched with a mixture of polyphenolic compounds, β-carotene, probiotics, and n-3 and n-6 polyunsaturated fatty acids. In total, *n* = 3,017 differentially expressed (DE) genes were identified, including *n* = 218 DE genes between control and BASE groups after 3 months of feeding and *n* = 1,262 after 14 months. BASE-diet influenced the expression of genes involved particularly in the gonadotrope cell activation pathway and guanylate cyclase pathway, as well as in mast cell activation, gap junction regulation, melanogenesis and apoptosis. Especially genes involved in regulation of GnRH were strongly affected by BASE-diet. This effect was stronger with the age of animals and the length of diet use. It may suggest a link between the diet, reproductive system function and aging.

## Introduction

The influence of diet on human health has been studied for a long time. In the fourth century BC, Hippocrates already stated: “Let food be your medicine and medicine be your food”. This theory was again recalled in the nineteenth century thanks to Ludwig Feuerbach who wrote “Man Is What He Eats”. Today’s scientific knowledge confirms the words of these great scientists. Recent studies have shown that our diet is an important factor which could contribute not only to the development but also to the inhibition of chronic diseases, including osteoporosis, diabetes, cancer, atherosclerosis, cardiovascular disease, neurodegenerative diseases and obesity (Virmani et al. 2006, 2013; Joseph et al. 2009; Lillycrop and Burdge 2012). It is possible due to nutritional factors, which may induce epigenetic changes also via direct influence on gene expression (Alam et al. 2012). In the late twentieth century, Nancy Fogg-Johnson and Alex Meroli created a new term which combines two fields of science: nutrition and genetics, named nutrigenomics. The aim of nutrigenomics is to study how various food ingredients affect the expression of specific genes and therefore provide tools to understand and control the worldwide epidemic of specific chronic diseases. It has been proven that these diseases more often arise from dysfunctional biological networks, instead of single common gene mutation (Liu et al. 2010; Ferguson et al. 2007a, b; Astley 2007).

Dietary interventions may induce changes in the metabolic and inflammatory state by modulating the expression of important genes involved in the chronic disorders (Lottenberg et al. 2012). This particularly applies to biologically active substances which have a protective influence on the body. For example, recent studies have shown that polyunsaturated fatty acids (PUFA) downregulate ATP-binding cassette transporter A-1 (ABCA-1), which results in reductions in HDL-C concentrations. PUFA suppress the liver X receptor/retinoid X receptor (LXR/RXR) gene responsible for ABCA-1 synthesis, which is a transporter involved in the HDL formation (Uehara et al. 2007). Furthermore, PUFA modulate expression of several genes involved in oxidative processes (such as PPAR-α), while impairing the sterol-regulatory element binding proteins (SREBPs) involved in lipogenesis (Hannah et al. 2001). A different study demonstrated that diet enriched with anti-inflammatory mixture containing resveratrol, green tea extract, α-tocopherol, vitamin C, omega-3 polyunsaturated fatty acids and tomato extract affected genes involved in inflammatory processes, oxidative stress and metabolism (Bakker et al. 2010). Also various dietary components including omega-3 fatty acids, plant flavonoids and carotenoids have been demonstrated to modulate gene expression, by decreasing iNOS and COX-2 gene expression induced by gliadin in RAW 264.7 macrophages stimulated with IFN-γ. Therefore, these compounds could preserve intestinal barrier integrity, play a protective role against toxicity of gliadin peptides and have a role in nutritional therapy of, for example, celiac disease (Ferretti et al. 2012; De Stefano et al. 2007).

Taking the latest data from the fields of nutrigenomics into consideration, the aim of the present study was to evaluate the influence of long-term (14 months) use of biologically active substances-enriched diet (BASE-diet) containing the mixture of polyphenolic compounds, β-carotene, probiotics and n-3 and n-6 polyunsaturated fatty acids on transcriptomic profile of rats liver. However, in the present study we did not want to investigate the mechanisms of action of the individual components, as these are already known. The main emphasis has been placed on the possible cumulative action of these compounds in the situation when they were added simultaneously as ingredients of semi-synthetic diet. Since 14-month period in case of rats is more than half of their life’s span, the obtained results could be of great value.

## Materials and methods

### Animal and diets

Experiment was carried out on thirty-six 8-week-old male Sprague–Dawley rats (Charles River Laboratories, Germany). The experiment was performed with the approval of the local ethical committee. Rats were kept in individual cages. Water and food were available ad libitum. The environment was regulated at 22 ± 0.5 °C, air humidity of 50 % on a 12 h/12 h L/D photoperiod throughout the entire experiment. The body weight and the feed consumption were recorded weekly. Animals were divided into two groups: a control group (*n* = 18) and an experimental group (*n* = 18).

Rats from both groups were receiving for 14 months semi-synthetic diets (control and BASE-diet) formulated according to the nutritional requirements for laboratory animals (NRC 1995) and are described in Table 1. Application of the semi-synthetic diet allowed the elimination of the additional impact of biologically active compounds contained in commercially available diets. BASE-diet was additionally enriched with the following biologically active compounds: 6 % of salmon fat replacing lard (to increase the level of unsaturated fatty acids), 8 % of hydrolysed water extract from small-leaved linden (*Tilia* *cordata*) inflorescence (as a source of antioxidant compounds), 8 % of puree from giant pumpkin (*Curcubita* *macima*) (the source of beta-carotene) and 1 % of two strains of bacteria with documented probiotic activity: *Lactobacillus* *acidophilus* LA-5 and *Bifidobacterium* *animals* ssp. *lactis*. The dry matter content of both diets was the same. Exact composition of macronutrients, micronutrients and vitamins mixtures included in the experimental diets is presented in Table 2.

**Table 1:** Composition of diets used for 14-month-long feeding of rats

	Control diet (g/kg diet)	BASE-diet (g/kg diet)
Rapeseed oil	10.0	20.0
Salmon fat	0.0	60.0
Lard	90.0	20.0
Hydrolysed water extract from linden (*Tilia cordata*) inflorescence	0.0	80.0
Puree from pumpkin (*Cucurbita maxima*)	0.0	80.0
Probiotic (LA5/BB12)	0.0	1.0
Mixture of macronutrients	36.3	36.3
Mixture of micronutrients	0.5	0.5
Mixture of vitamins	10.0	10.0
l-methionine	2.2	2.2
Casein	200.0	200.0
Wheat starch	482.0	465.0
Potato starch	10.0	10.0
Water	144.0	0.0
Saline	15.0	15.0

**Table 2:** Composition of the mixtures of macronutrients, micronutrients and vitamins used in control and BASE-diet fed to rats for 14 months

Macronutrients mixture composition (g/kg of mix)	
CaHPO_4_ × 2H_2_O	27.89
K_2_HPO_4_	2.43
NaCl	0.92
K_2_SO_4_	2.04
CaCO_3_	0.63
Na_2_HPO_4_ × 12H_2_O	1.61
MgO	0.75
Micronutrients mixture composition (g/kg of mix)	
C_3_H_4_(OH)(COO)_3_Fe x 3H_2_O	3.76
Zn(CH_3_COO)_2_ × 2H_2_O	0.79
MnCO_3_	2.34
Cu(CH_3_COO)_2_ × H_2_O	0.5
KJ	0.004
C_3_H_4_(OH)(COOH)_3_	*ad* 100 g
Vitamins mixture composition (mg/kg of mix)	
Vitamin A	0.69
Vitamin D_3_	0.5
Vitamin E	98.2
Para-aminobenzoic acid	100.0
Inositol	100.0
Niacin	40.0
Ca-pantothenate	40.0
Vitamin B_2_	8.0
Vitamin B_1_	5.0
Vitamin B_6_	5.0

The content of biologically active substances in feed was measured as follows. The fatty acid content was determined both in the raw ingredients and in the feed samples using gas chromatography (Table 3). Quantitative analysis of the total polyphenolic compounds in the hydrolysed water extract from small-leaved linden inflorescence was made using the method with Folin reagent (1 N) in the presence of Na_2_CO_3_ (20 %). The analysis of beta-carotene and other carotenoids content was performed using high-performance liquid chromatography coupled with electrochemical detection (HPLC–ECD).

**Table 3:** The fatty acid profiles in control and BASE-diets

Fatty acids	Control diet	BASE-diet
Saturated (%)	32.70	17.24
Monounsaturated (%)	32.44	30.75
Polyunsaturated (%)	10.43	18.32
20:5 n-3 (%)	0.02	2.10
22:6 n-3 (%)	0.02	3.60
Sum of n-3 fatty acids (%)	2.90	9.02
Sum of n-6 fatty acids (%)	7.54	9.42

After 3 and 14 months of experiment, animals from each experimental group (*n* = 9) were euthanized by exsanguination under general anaesthesia with the isoflurane. Liver samples from six randomly selected rats from each group were taken.

### Microarrays

Liver samples were frozen with liquid nitrogen immediately after collection and stored at −80 °C until extraction. Total RNA was extracted with RNeasy Lipid Tissue Mini Kit (Qiagen, Germany) following the manufacturer’s recommended protocol. Subsequently, potential genomic DNA contamination was eliminated using Deoxyribonuclease I Amplification Grade (Sigma, USA) and RNeasyMinElute Cleanup Kit (Qiagen, Germany). RNA quantity and quality was measured using NanoDrop 2000 (NanoDrop Technologies, USA) and Bioanalyzer (Agilent Technologies, USA). To ensure optimal data quality, only RNA samples with RIN number ≥8.8 were included in the analysis.

The analysis of gene expression profile was performed using SurePrint G3 Rat Gene Expression Microarray, 8 × 60 K (Agilent Technologies, USA). The Low Input Quick Amp Labeling Kits (Agilent, USA) was used to amplify and label target RNA to generate complementary RNA (cRNA) for oligo microarrays used in gene expression profiling. Experiment was performed using a common reference design, where the common reference was RNA from 10-week-old healthy rats not participating in the experiment, housed for 2 weeks in the same room with experimental rats. On each two-colour microarray, 300 ng of cRNA from the control rats (labelled by Cy3) and 300 ng of cRNA from the rats fed with BASE-diet (labelled by Cy5) were hybridized. Microarray hybridization was performed with the Gene Expression Hybridization Kit (Agilent Technologies, USA), according to the manufacturer’s protocols. RNA Spike In Kit (Agilent Technologies, USA) was used as an internal control. In total, 24 microarrays, one for each animal, were done. Acquisition and analysis of hybridization intensities were performed using an Agilent DNA microarray scanner. The final analysis was carried out on 22 microarrays, which passed through the Agilent Feature Extraction’s and the Gene Spring’s control (one microarrays from the control group after 3 months of experiment and one from the experimental group after 3 months of experiment were rejected due to insufficient quality).

### Signal detection and statistical analysis

Data were extracted and background was subtracted using the standard procedures contained in the Agilent Feature Extraction (FE) Software version 10.7.3.1. FE performs also Lowess normalization. The statistical analysis was performed using Gene Spring 12 software (Agilent, USA). The samples underwent quality control and the results showed that each sample had similar QC metric profile. The next step was filtering probe sets by flags to remove poor-quality probes (absent flags). The statistical significance of the differences was evaluated using one-way ANOVA and Tukey’s HSD post hoc test (*p* < 0.05). A multiple testing correction was performed using Benjamini and Hochberg False Discovery Rate (FDR) <5 %. Microarray data were deposited at the Gene Expression Omnibus data repository under the number GSE51657 according to the MIAME requirements.

To identify the list of signalling pathways, the microarray data were analysed using Pathway Studio 6.0 (Ariadne Genomics).

### Real-time PCR

To verify microarray results, the expressions of three randomly selected genes (GPX1, IRF7 and PRODH) were measured using real-time PCR method. The sequences of these genes were obtained from Ensembl database. Primers were designed using Primer-Blast software (NCBI database) and then checked for secondary structures using Oligo Calculator (free on-line access). The secondary structures of the amplicon were examined using m-fold Web Server (free on-line access). As a house keeping gene ACTB were used (Huang et al. 2013; Wang et al. 2013). The sequences of the primers are listed in Table 4.

**Table 4:** Primer sequences for real-time PCR verification of microarray results

Gene’s name	NM number	Sense primer (5′–3′)	Antisense primer (5′–3′)
Tested genes			
GPX1	NM_030826.2	CCTAAGGCATTCCTGGTATC	CCATCTGAGGGGATTTTTCT
IRF7	NM_001033691.1	GTCTAGCACCAATAGTCTCTAC	AAGGTCCACTAGAGATGACA
PRODH	NM_001135778.1	CACAGGTGCCTTAACTATGTTC	TAACTCCTTCATCCTGCACAAC
Reference gene			
ACTB	NM_031144.3	CCCACACTGTGCCCATCTAT	AAGGGTGTAAAACGCAGCTC

cDNA was synthesized using Enhanced Avian HS RT-PCR Kit (Sigma-Aldrich, St. Louis, Missouri). All analyses were performed on individual samples of total RNA using a Brilliant III Ultra-Fast SYBR Green QPCR Master Mix kit (Agilent Technologies, USA) following the manufacturer’s protocol. Each sample was tested 3 times in a Stratagene Mx3005P Quantitative PCR instrument for RT-PCR. The relative expression of the target gene was calculated according to the following formula:

$$\Delta \varDelta {\text{CT}} = \Delta {\text{CT}}\left( {\text{sample}} \right) - \Delta {\text{CT}}\left( {\text{normal}} \right)$$

where ΔCT is the difference in CT between the targeted gene and housekeeping controls by minimizing the average CT of the controls. The fold-change calculated as: 2^−ΔΔCT^ (Livak and Schmittgen 2001).

## Results

**Fig. 1:**
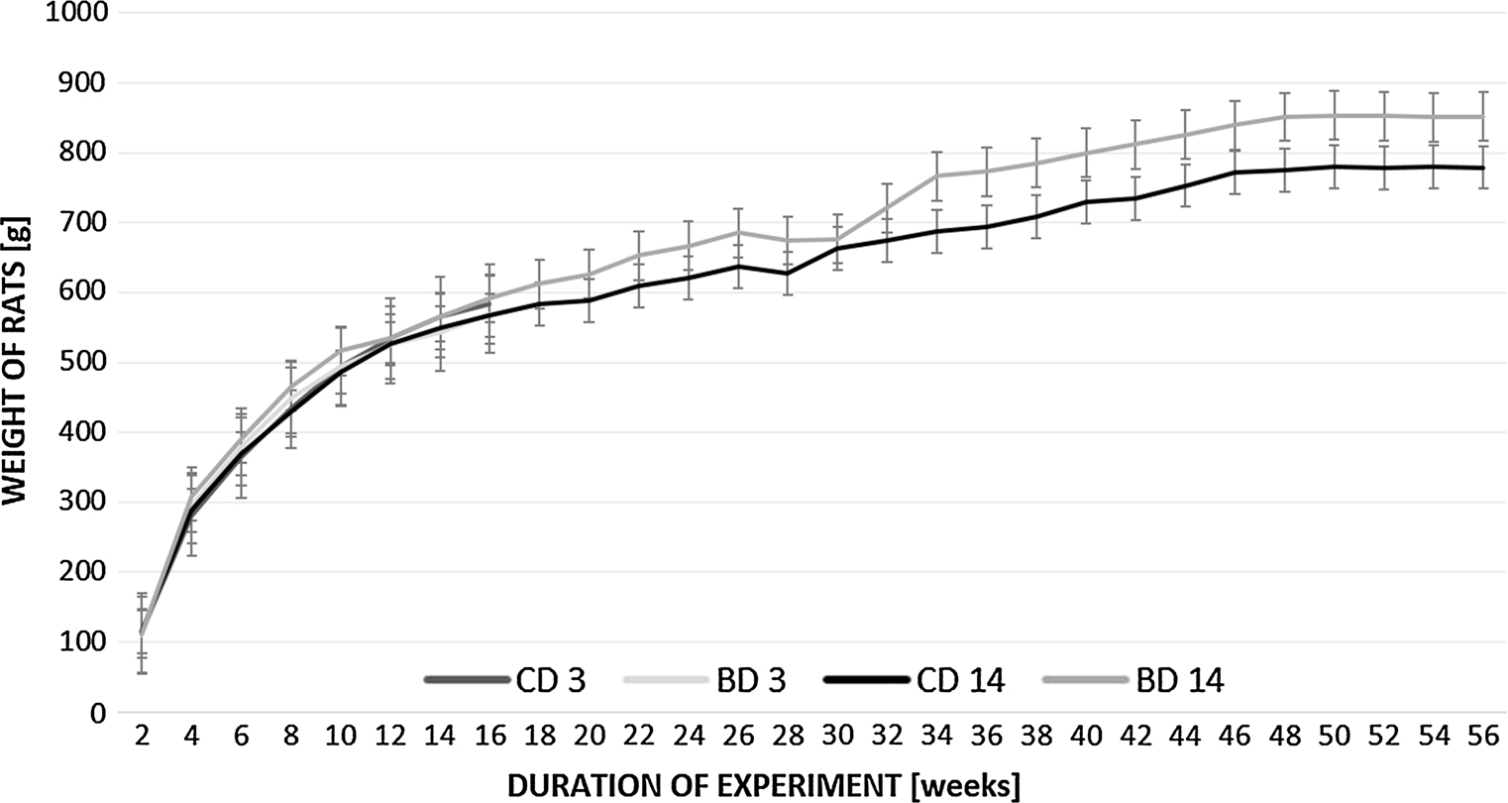
Weight of rats participating in the experiment. CD 3—rats from control group after 3 months of experiment; BD 3—rats from BASE group after 3 months of experiment; CD 14—rats from control group after 14 months of experiment; BD 14—rats from BASE group after 14 months of experiment

**Fig. 2:**
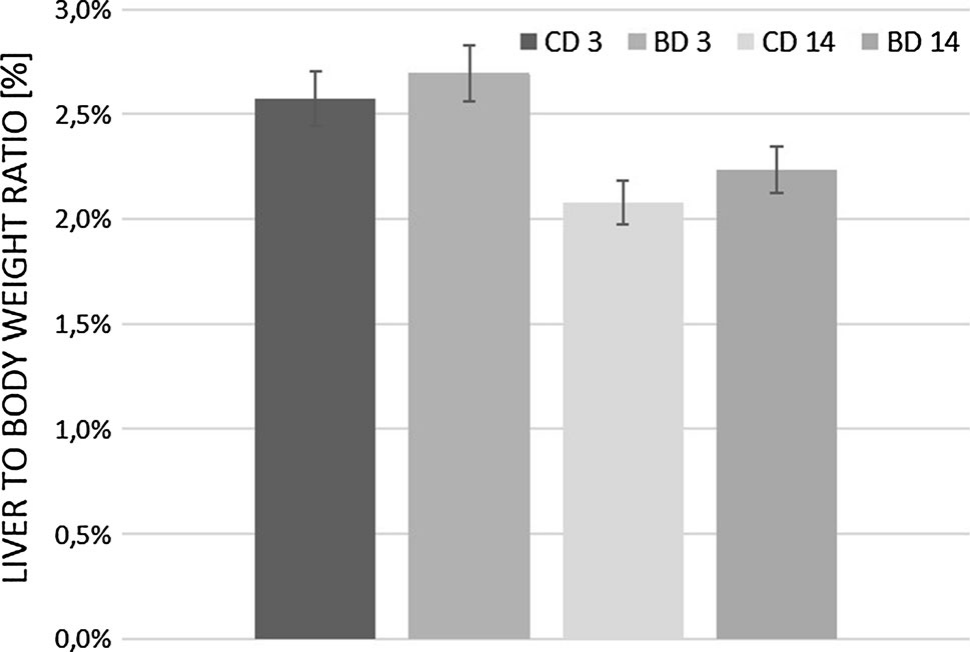
Liver to body weight ratio (%). CD 3—rats from control group after 3 months of experiment; BD 3—rats from BASE group after 3 months of experiment; CD 14—rats from control group after 14 months of experiment; BD 14—rats from BASE group after 14 months of experiment

**Fig. 3:**
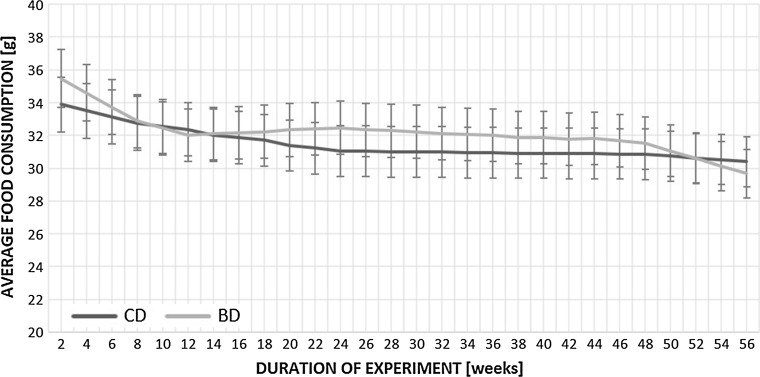
Average food consumption [g] of rats participating in the experiment. CD—rats from control group; BD—rats from BASE group

There was no difference in initial body weight and liver to body weight ratio among all experimental groups. Over the course of the 14-month study, all animals gained body weight in a time-dependent manner, regardless the treatment (Figs. 1, 2). There was also no difference in the amount of diet eaten between control and experimental rats (Fig. 3). Earlier published data showed that there were no statistically significant differences between control and BASE-diet groups in blood morphology parameters as well as biochemical liver function parameters (Oszkiel et al. 2014).

**Fig. 4:**
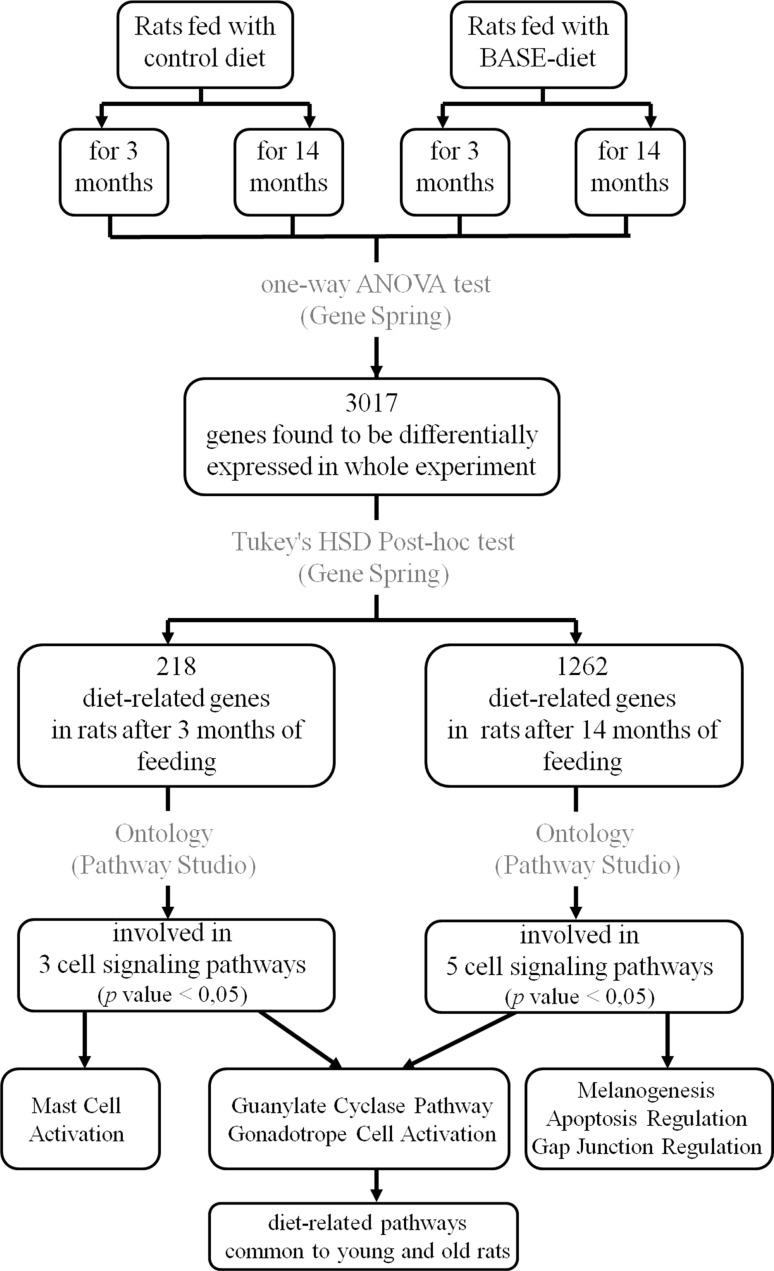
The analysis diagram of the data obtained in the experiment

The consecutive steps of microarray data analysis have been presented in Fig. 4. Analysis started from identification of all differentially expressed (DE) genes in whole experiment and was conducted using ANOVA in Gene Spring software. In total, *n* = 3,017 DE genes were identified. Then, using Tukey’s HSD post hoc test the lists of differentially expressed genes between experimental groups were identified (Table 5). This analysis revealed *n* = 218 differentially expressed genes between control and BASE groups after 3 months of feeding, and *n* = 1,262 differentially expressed genes between control and BASE after 14 months of feeding. These two sets of differentially expressed genes were considered as diet-induced genes since the differences in expression resulted only from the diet fed to the animals.

**Table 5:** The number of differentially regulated genes between each experimental group (total number of differentially regulated genes *n* = 3,017)

Group name^a^	CD 3	BD 3	CD 14	BD 14
CD 3	3,017	218	282	2,264
BD 3	–	3,017	142	2,255
CD 14	–	–	3,017	1,262
BD 14	–	–	**–**	3,017

^a^CD 3—rats from control group after 3 months of experiment; BD 3—rats from BASE group after 3 months of experiment; CD 14—rats from control group after 14 months of experiment; BD 14—rats from BASE group after 14 months of experiment

Each of the two data sets was analysed using Pathway Studio 6.0 (Ariadne Genomics) software in order to identify statistically significant Ariadne Cell Signalling Pathways in which differentially expressed genes were involved (based on Gene Ontology). After 3 months of BASE-diet feeding, three Ariadne Cell Signalling Pathways were significantly regulated (*p* value <0.05) (Table 6). After 14 months of BASE-diet feeding, five Ariadne Cell Signalling Pathways were significantly regulated (*p* value <0.05) (Table 7). There were two signalling pathways which were regulated both after 3 and 14 months of BASE-diet administration, namely guanylate cyclase pathway and gonadotrope cell activation pathway.

**Table 6:** The list of Ariadne Cell Signalling Pathways significantly regulated in rats liver after 3 months of BASE-diet feeding

Name	Type	Total entities	Expanded # of entities	Overlap	Percent overlap	Overlapping entities	*p* value
Mast cell activation	Pathway	64	558	9	1	SHC4, GIPC1, CYP4A11, MAPK12, HIP1, ELK1, PPP3CB, CYP2B6, DHRS4	0.02204
Guanylate cyclase pathway	Pathway	36	1, 219	15	1	SLC23A3, VASP, GIPC1, SLC5A5, TRPV5, SLC28A2, SLC24A4, PRKACA, DNMT3A, HIP1, ANP32A, DCX, SPTBN1, EML2, NRM	0.0308
Gonadotrope cell activation	Pathway	71	728	10	1	SHC4, GIPC1, CYP4A11, PRKACA, MAPK12, HIP1, ELK1, PPP3CB, CYP2B6, DHRS4	0.04255

**Table 7:** The list of Ariadne Cell Signalling Pathways significantly regulated in rats liver after 14 months of BASE-diet feeding

Name	Type	Total entities	Expanded # of entities	Overlap	Percent overlap	Overlapping entities	*p* value
Gap junction regulation	Pathway	51	661	36	5	NRG1, PMCH, CCL20, ERBB2, PRKG1, DRD2, AGRP, GALP, PENK, HRH1, CGA, CNR1, FGF19, NPY5R, FFAR1, CCKBR, OPRL1, OPRK1, UCN3, FGF5, MC2R, HTR2C, NPSR1, RLN1, PRKG2, FGF22, NRG2, VGF, SSTR1, GRM3, PTH2R, GRM7, GJB3, RASGRF2, SHC3, GPR139	0.00019
Melanogenesis	Pathway	50	694	32	4	NRG1, PMCH, CCL20, ERBB2, DRD2, CYP1B1, AGRP, WNT10B, GALP, PENK, FGF19, NPY5R, OPRL1, UCN3, FGF5, MC2R, LRP5, DCT, RLN1, TYRP1, WNT11, NDP, FGF22, NRG2, VGF, SSTR1, WNT16, GRM3, GRM7, SHC3, DZIP3, ECH1	0.00682
Guanylate cyclase pathway	Pathway	36	1, 219	49	4	GABRG3, KRT82, AIF1L, KRT26, GLRA4, Rims2, SLC12A5, SLC26A4, P2RX2, KCND2, KCNJ6, CLCN1, ATP12A, SCN2A, NOS1, PRKG1, TRPM5, TRPV3, TRPM3, NPPC, ANK2, TRPA1, MYBPC3, SCN10A, SLC1A1, SLC1A2, SLC34A3, KALRN, SLC28A3, SCN3A, PRKG2, SUV39H1, SUV39H2, GRIA1, DISC1, TRPC7, KCNJ13, KRT34, TUBE1, KCNIP1, ESPN, KCNC2, GABRA2, CAPZA3, KCNE1L, KRT31, ATP1A4, SLC6A15, TEKT3	0.01162
Gonadotrope cell activation	Pathway	71	728	32	4	Rims2, NRG1, PMCH, ERBB2, GALP, PENK, CGA, FGF19, UCN3, FGF5, CATSPER4, POU1F1, NPSR1, RLN1, EHF, FGF22, NRG2, POU4F1, VGF, ARHGEF7, PTH2R, CACNA1B, LHX4, RASGRF2, SHC3, CACNA2D4, LHX6, PPP3R2, ETV2, CACNG3, POU6F2, CACNB1	0.01346
Apoptosis regulation	Pathway	69	624	26	4	NXPH2, IL4, P2RX2, TRPV3, TRPM3, TGFB2, TRPA1, IL21, NTRK2, IL6ST, CATSPER4, TNFRSF18, TNFSF18, IL9R, IL5RA, CNTFR, CACNA1B, TRPC7, OMG, IL1F5, CACNA2D4, PPP3R2, IFNA16, IL28RA, CACNG3, CACNB1	0.04523

**Fig. 5:**
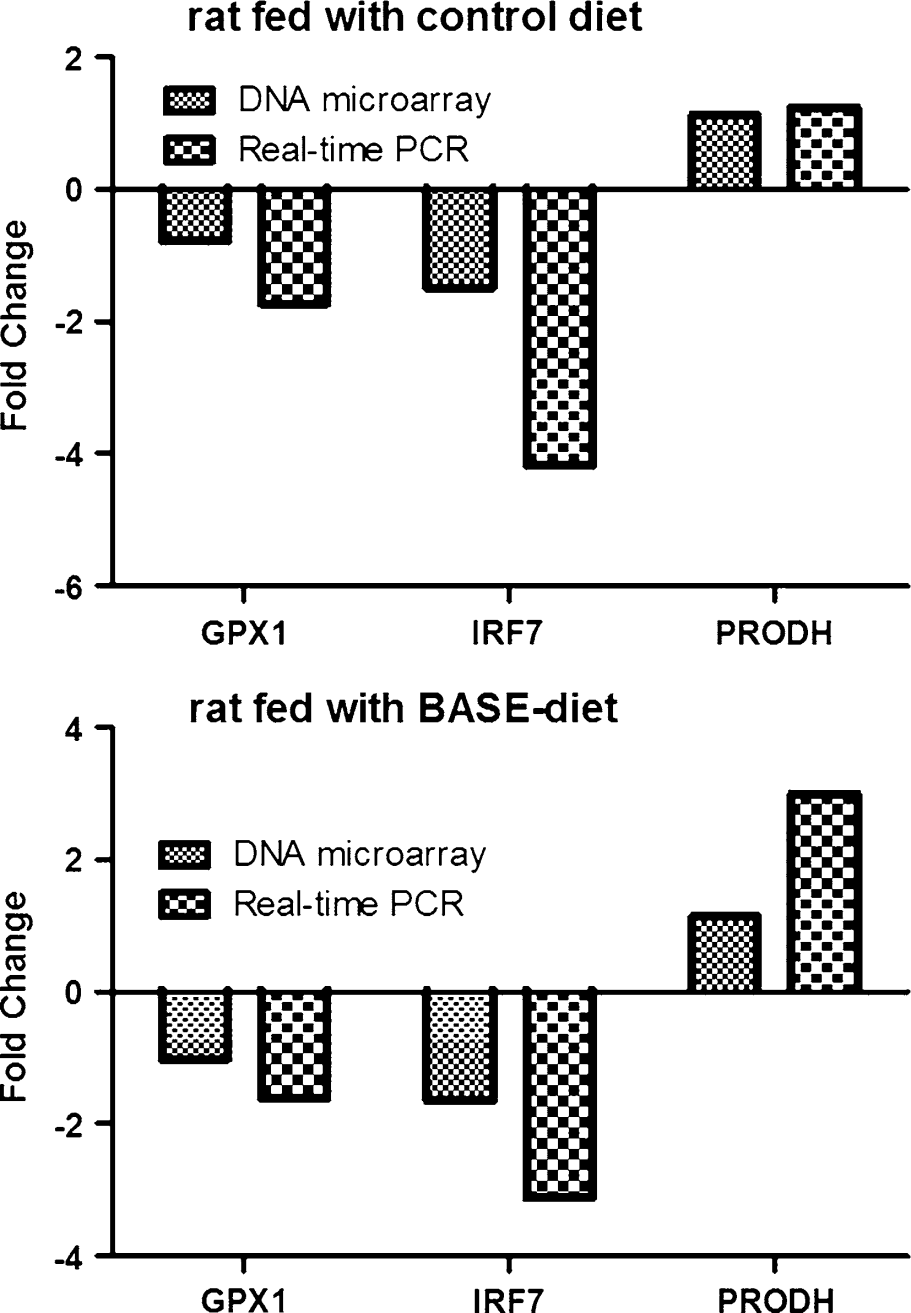
Expression of *GPX1*, *IRF1* and *PRODH* genes in livers of control rats and rats fed with BASE-diet measured using DNA microarrays and real-time PCR (normalized vs. ACTB)

Obtained microarray results were also validated using real-time PCR on randomly selected three genes. As shown in Fig. 5, gene expression for GPX1, IRF7, PRODH matched the expression obtained from microarray analysis.

## Discussion

In the present study, we investigated the influence of biologically active substances-enriched diet (BASE-diet) fed for a long period of time (14 months) on the transcriptome of rat liver. It means that two factors could influence the results—the experimental diet and age of rats. Based on the numbers of genes regulated in individual comparisons (adult vs. older; control diet vs. BASE-diet), we could conclude that age factor was responsible for the regulation of higher number of genes (number of differentially regulated genes between adult and old BASE-diet fed rats was *n* = 2,255) than the diet factor (number of differentially regulated genes between control and BASE-diet fed rats was *n* = 218 after 3 months of experiment and *n* = 1,262 after 14 months of experiment) (see Table 5).

**Fig. 6:**
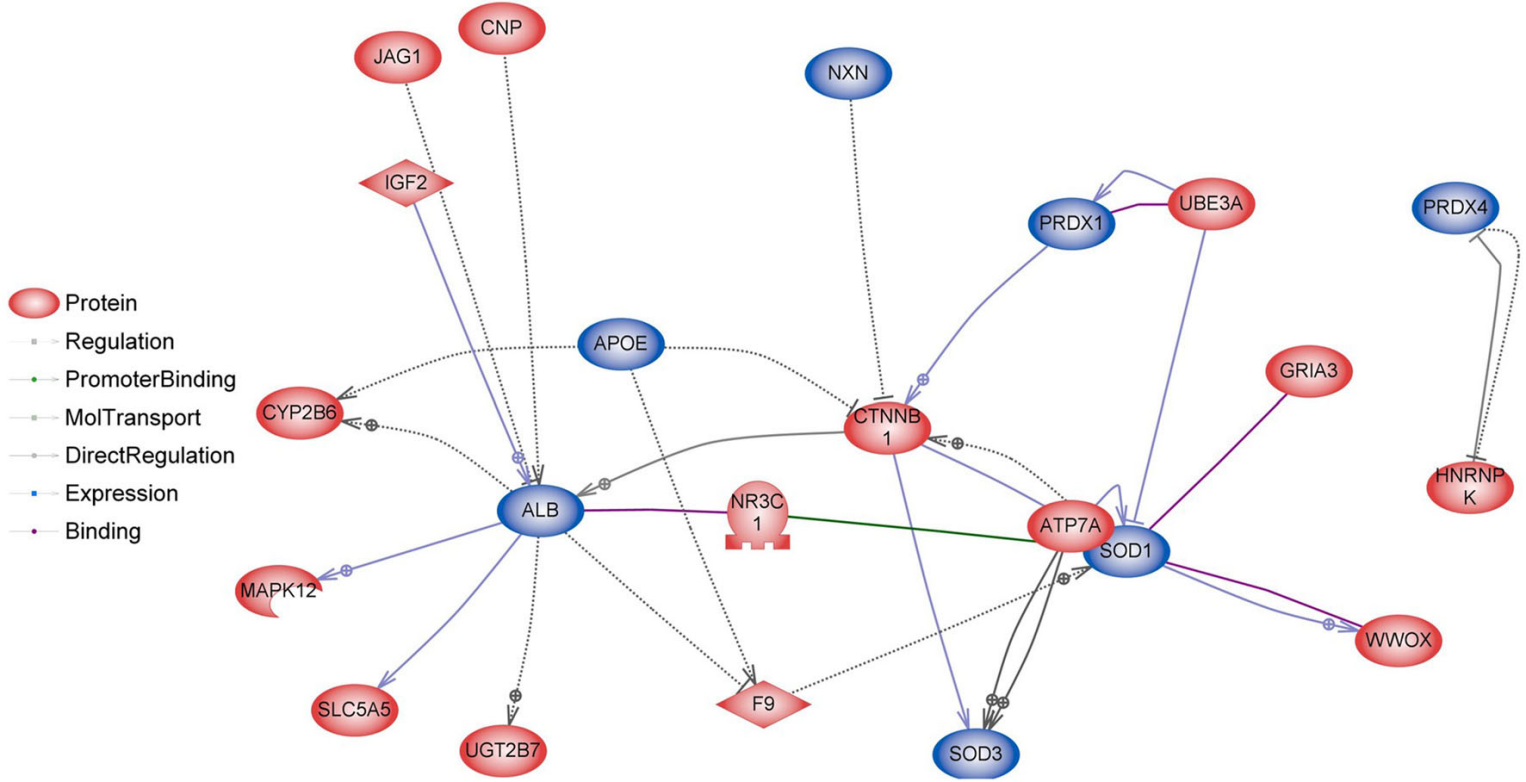
Interactions between proteins involved in antioxidant activity (*blue*) and proteins encoded by differentially regulated genes (*red*) in livers of rats after 3 months of BASE-diet feeding

**Fig. 7:**
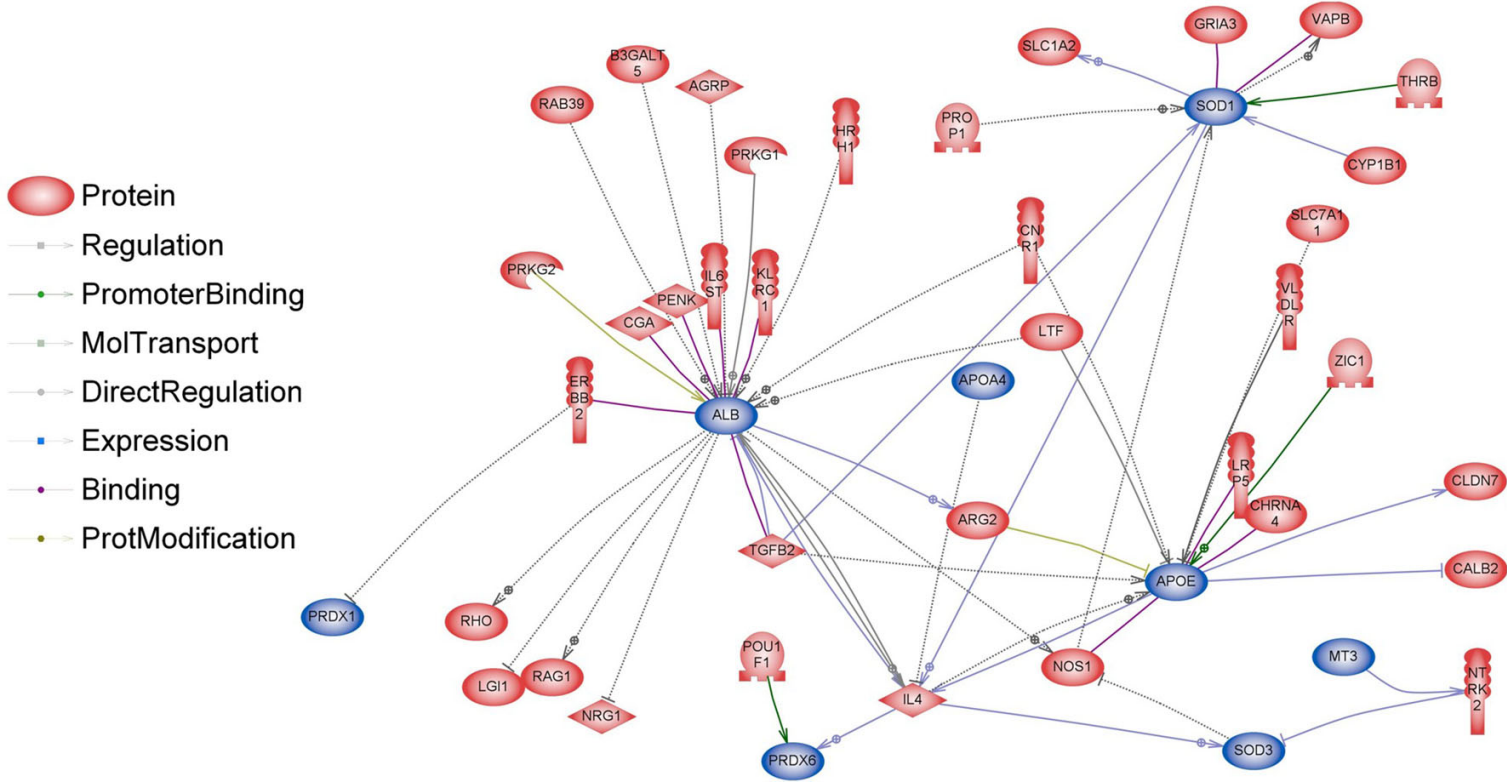
Interactions between proteins involved in antioxidant activity (*blue*) and proteins encoded by differentially regulated genes (*red*) in livers of rats after 14 months of BASE-diet feeding

Our previous study has shown that BASE-diet, which was enriched with the mixture of polyphenols, beta-carotene, probiotics and polyunsaturated fatty acid, prevented from hepatic and systemic oxidative damage and was able to attenuate the development of some senile features in adult and old rats (Oszkiel et al. 2014). In rats fed with BASE-diet, the GSH/GSSG ratio has increased, GSH-Px and GSSG-R activities decreased, and SOD activity decreased when compared to control animals. These results suggest that BASE-diet positively influenced some parameters of antioxidant defence within the body. Because of that we investigated whether differentially regulated genes were involved in antioxidant activity. Analyses performed in Pathway Studio Software show relations between proteins encoded by genes involved in antioxidant activity and proteins encoded by genes differentially regulated in livers of rats fed with BASE-diet (Figs. 6, 7). It is clearly visible that in older rats the number of differentially regulated genes involved in antioxidant activity is significantly higher when compared to adult rats.

As expected, we were able to identify differentially expressed genes between control and BASE-diet both in adult (after 3 months of feeding) and older rats (after 14 months of feeding). Significant difference in the expression of these genes resulted in significant regulation of a few signalling pathways (three in the case of adult rats and five in the case of older rats). Two of these pathways were the same in adult and older rats, so we concluded that these two were purely dependent on the diet.

BASE-diet significantly influenced the expression of gonadotrope cell activation pathway and guanylate cyclase pathway. Gonadotrope cell activation pathway is involved in regulation of the reproduction system. It regulates the activity of the basophilic cells of the anterior pituitary gland specialized in secreting follicle-stimulating hormone (FSH) or luteinizing hormone (LH). FSH and LH are released by a trophic peptide hormone—gonadotropin-releasing hormone (GnRH). FSH stimulates development of ovarian follicles and regulates spermatogenesis in the testis. LH causes ovulation and formation of the corpus luteum in the ovary, stimulates production of oestrogen and progesterone by the ovary and stimulates testosterone production by the testis (Guyton and Hall 2006; Okamura et al. 2013). Previous studies reported that reproductive activity can be directly influenced by nutrients, including the biologically active compounds. Among such compounds are polyphenols which were included into BASE-diet. Chen et al. (2010) indicate that genistein and resveratrol can increase the ovarian follicular reserve and prolong the ovarian lifespan in rats. Gebrie et al. (2005) proved that the extract of *Rumex steudelii* (Ethiopian plant which roots contain phytosterols and polyphenols) prolonged significantly the oestrus cycle and the dioestrous phase. Studies of Colitti et al. (2007) revealed that polyphenols can protect from stress which activates the hypothalamo–pituitary–adrenal axis leading to enhanced glucocorticoid secretion and concurrently disrupts ovarian cycle. Thereby, polyphenols can improve reproduction activity (Matteri et al. 2001; Zhang et al. 2006; Colitti and Stefanon 2006; Sgorlon et al. 2006; Colitti et al. 2007).

Gonadotrope cell activation pathway could also be regulated by β-carotene. β-carotene is not only a retinol precursor, but also fulfils similarly function to vitamin E. It is a free radical scavenger, acting especially on singlet oxygen, and thus it is a potent antioxidant, similarly to other carotenoids. Moreover, many gene products linked to reproduction can be modulated by the product of retinol oxidation—retinoic acid. Therefore, it is considered that an optimal intake of β-carotene has a positive effect on fertility (Schweigert et al. 2003; Arechiga et al. 1998). Recent study has shown a direct relationship between β-carotene concentration at ovarian level and size and progesterone secretion by *corpora lutea*. It is suggested that β-carotene may have a positive effect on luteogenesis and luteal activity (Haliloglu et al. 2002; Arellano-Rodriguez et al. 2009).

Also the fat content in the diet affects the reproductive system. Study carried out on cows shows that fat supplementation is associated with increased dominant follicle diameter, greater progesterone concentrations, modulation of prostaglandin synthesis and improved oocyte and embryo quality. It collectively results in increasing likelihood of conception (Garcia-Bojalil et al. 1998; Lucy et al. 1993; Mattos et al. 2002).

**Fig. 8:**
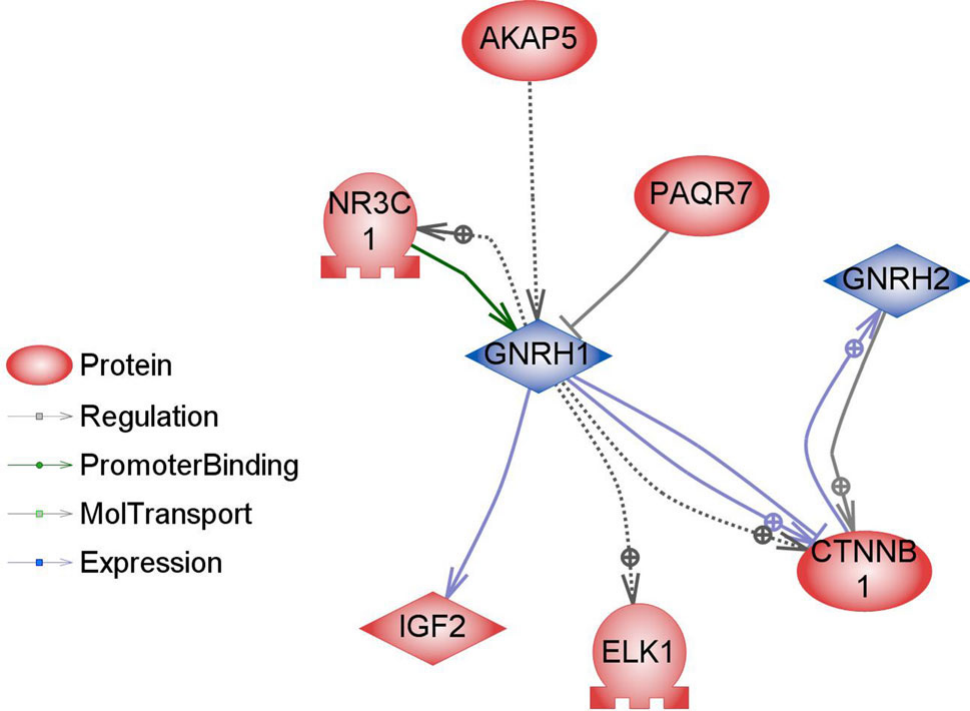
Interactions between GnRH-I and GnRH-II (*blue*) and proteins encoded by differentially regulated genes (*red*) in livers of rats after 3 months of BASE-diet feeding

**Fig. 9:**
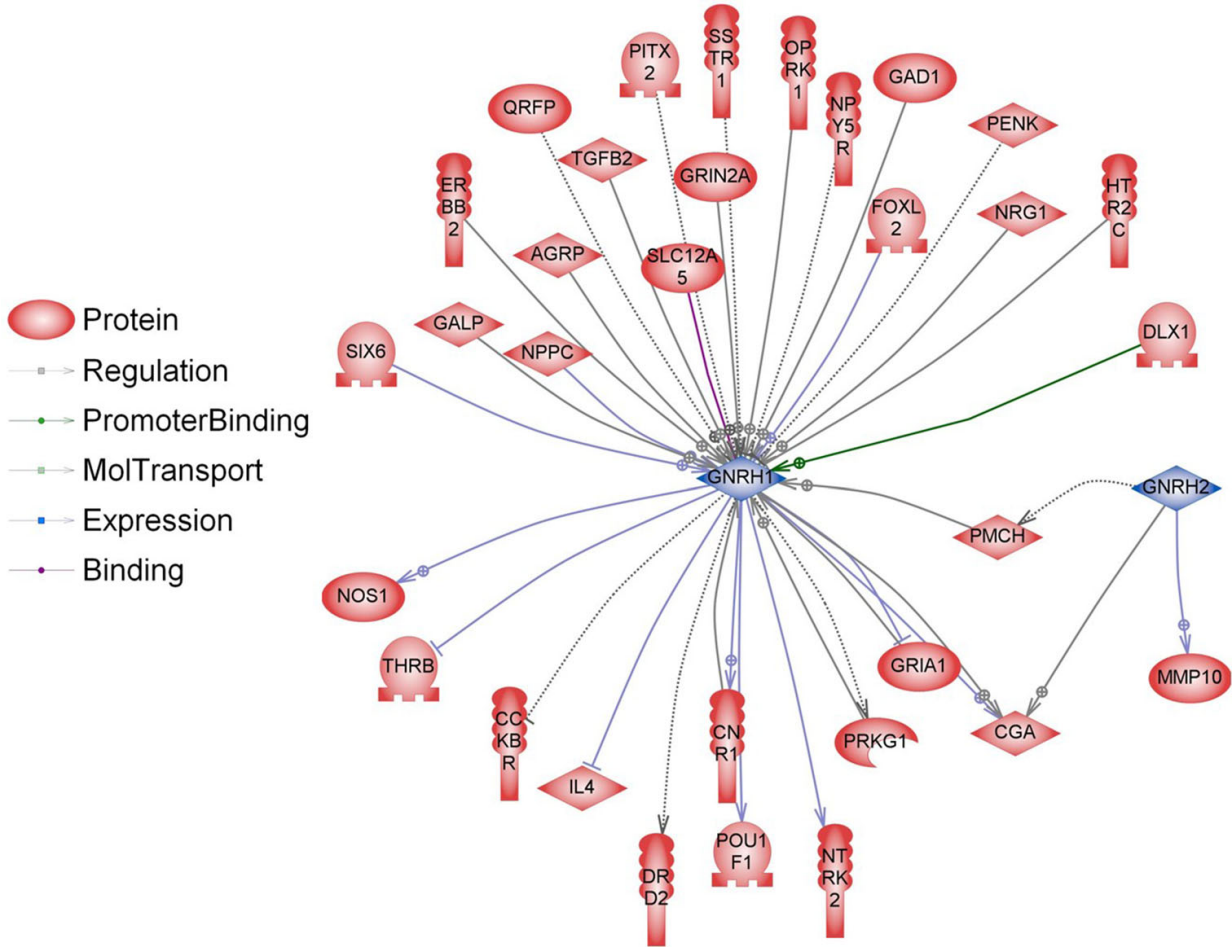
Interactions between GnRH-I and GnRH-II (*blue*) and proteins encoded by differentially regulated genes (*red*) in livers of rats after 14 months of BASE-diet feeding

**Fig. 10:**
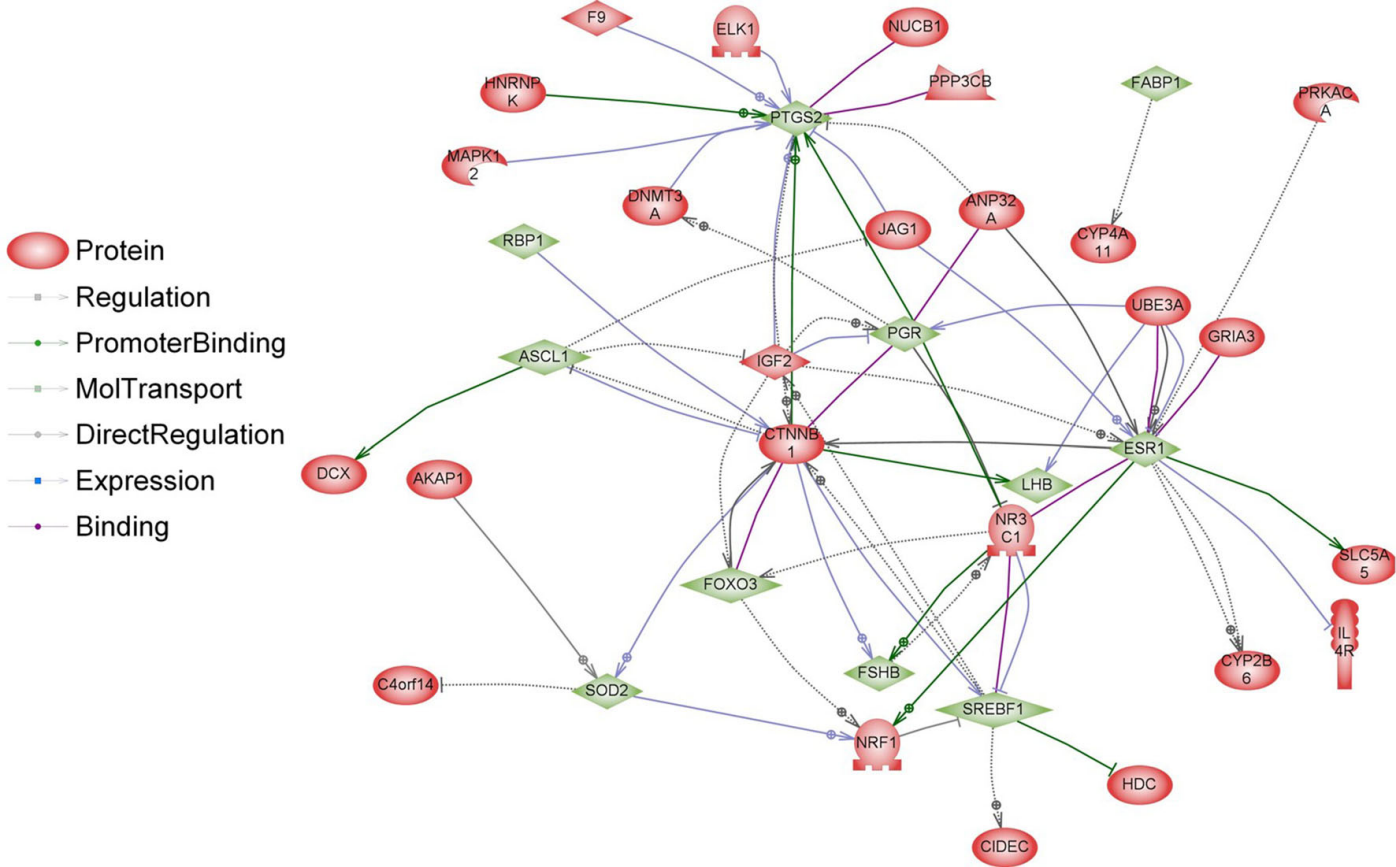
Interactions between “food interventions-related proteins” involved in reproductive system regulation (*green*) and proteins encoded by differentially regulated genes (*red*) in livers of rats after 3 months of BASE-diet feeding

**Fig. 11:**
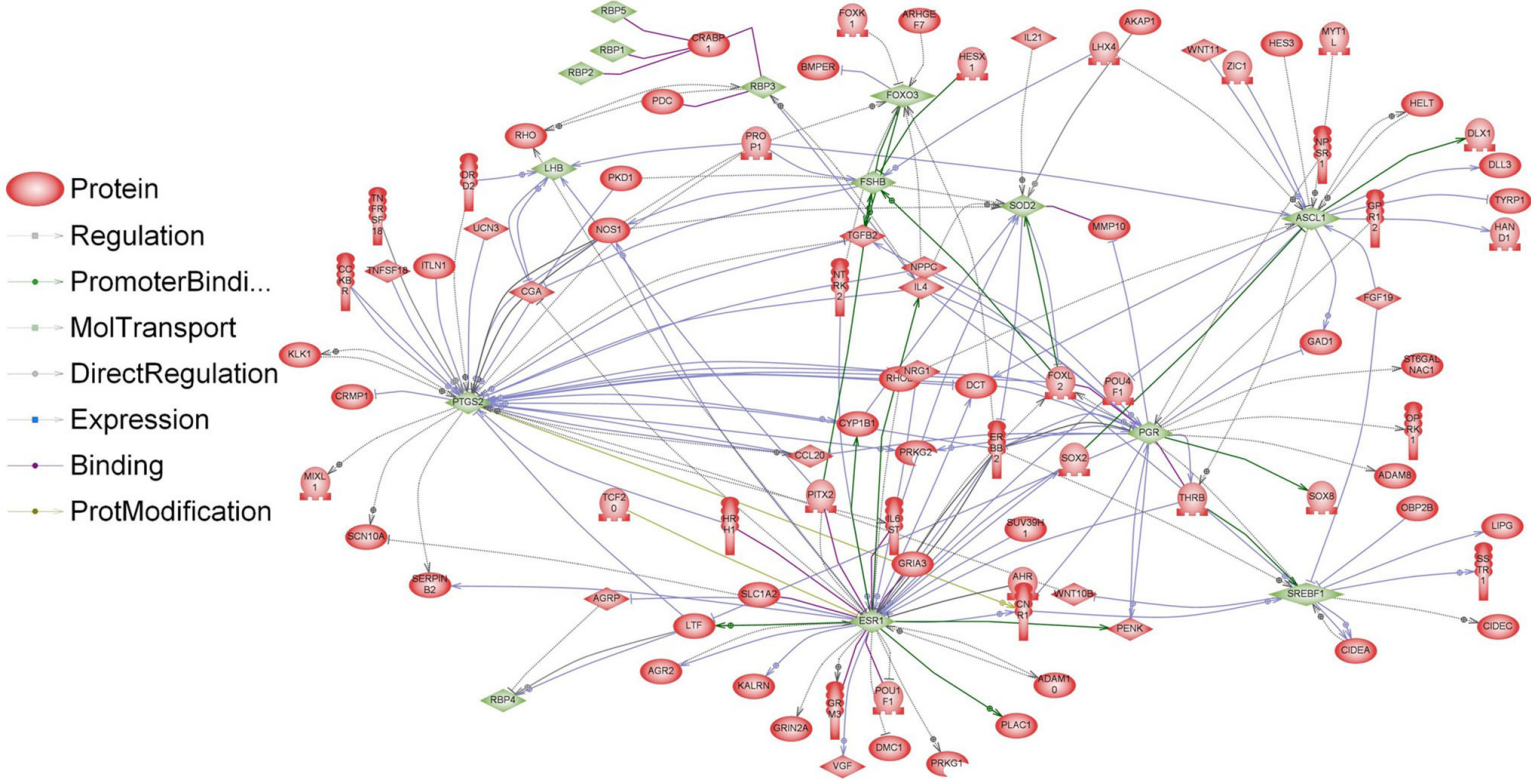
Interactions between “food interventions-related proteins” involved in reproductive system regulation (*green*) and proteins encoded by differentially regulated genes (*red*) in livers of rats after 14 months of BASE-diet feeding

However, there is some evidence that diet affects the reproductive system also on the transcriptome level. Majority of the studies focusing on transcriptional regulation of GnRH genes concerned GnRH-I; however, recently also a study concerning GnHR-II has been published (Lee et al. 2008). In our study, GnRH-I and GnRH-II genes, which are expressed mostly in the brain, were not directly regulated. However, we identified 38 genes involved in indirect regulation of GnRH-I and GnRH-II expression (Figs. 8, 9). The expression of some genes involved in regulation of GnRH can be modulated by food interventions. Plant polyphenols can alter the expression levels of oestrous cycle genes encoding PGHS-2 (upregulation), SOD2 and FOXO3 (downregulation) (Colitti et al. 2007). Dietary β-carotene can influence reproductive system on the transcriptome level as well. β-carotene is a precursor (inactive form) of vitamin A. It is currently believed that vitamin A regulates gene transcription also by retinol-binding proteins (RBP). Expression of the RBP genes is dependent on progesterone, which is dominant ovarian steroid hormone, known to be involved in the regulation of gonadotropin secretion. Progesterone regulates the GnRH-I gene through a feedback mechanism (Van Arnum 1998; Berry et al. 2011; Costello et al. 2010; Lee et al. 2008). Moreover, it has been demonstrated that unsaturated fatty acids may influence reproduction directly interfering with basal and GnRH-dependent gonadotrope activity (Garrel et al. 2011). Diet enriched with fish oil rich in 20:5 and 22:6 n-3 fatty acids modulate the hepatic expression of genes influencing reproductive performance: SREBF1, ASCL1 and FABP1 (Hutchinson et al. 2012). Dietary n-3 fatty acids also increase the progesterone receptor (PR) mRNA and oestrogen receptor-alpha (ER-1) expression (Bilby et al. 2006). Additionally, free fatty acids might directly modulate pituitary gonadotropin production by upregulating Lhb mRNA expression and suppressing Fshb mRNA expression (Sharma et al. 2013). None of the above-mentioned genes have been directly regulated in our experiment; however, a significant part of differentially expressed genes in the livers of BASE-diet fed rats was involved in regulation of all above-mentioned food-interventions-related proteins (Figs. 10, 11).

However, there are some latest data indicating that gonadotrope cell activation pathway gene could be expressed not only in reproductive tissues (Millar 2005). So far, the only well-known pathway connected with GnRH action was associated with fertility regulation in the hypothalamic–pituitary–gonadal axis. However, current research showed also that expression of genes involved in regulation of gonadotrope cell activation pathway was observed in other cells such as embryonic stem cell, adult brain, skeletal muscle, myocardium tissues, breast cancer stem cells, prostate cancer cell lines, pancreatic ductal adenocarcinoma, liver, kidney, thyroid cancer cells, mammary gland, prostate cancer, bone matrix and neuroblastoma cells (Ames et al. 2013; Chaudhry et al. 2013; Datta et al. 2012; Gandhi et al. 2013; Jones et al. 2007; Kim et al. 2014; Li et al. 2013; Ma et al. 2014; Polovlkova et al. 2013; Rosen et al. 2013; Savas et al. 2009; Singer et al. 2012; Turco et al. 2012; Zhang et al. 2009). Moreover, the latest studies demonstrate that GnRH are associated with regulation of proliferation, angiogenesis and inflammatory response, which are important in the pathogenesis of diseases associated with aging (Pincas et al. 2014). There are also some data about the negative relation between GnRH receptors and growth factors, which play a part in aging processes related to activity of stem cells, tissue regeneration, protein homeostasis and regulation of inflammatory processes leading to the age-related diseases (Cheung and Wong 2008). So far, there are only few reports about the influence of GnRH on the aging process and aging-associated diseases. De Haes et al. (2013) showed that silencing of the gene encoding the GnRH receptor resulted in prolongation of *C. elegans* life. In addition, Zhang et al. (2013) pointed out the relationship between the pituitary GnRH and immunoaging.

The second pathway which was influenced by BASE-diet is guanylate cyclase pathway. Guanylyl cyclase, in response to calcium levels, synthesizes 3′,5′-cyclic guanosine monophosphate (cGMP) from guanosine triphosphate (GTP). cGMP is associated with processes, such as phototransduction, circadian entrainment, olfactory transduction, vascular smooth muscle contraction, gap junction, long-term depression, salivary secretion, borderline and spontaneous hypertension, platelet activation and learning ability (Cornilescu et al. 2014; Arshavsky and Wensel 2013; Chae et al. 2007; Chen et al. 2007; Golombek et al. 2003; Wang et al. 2013; Dismuke et al. 2013; Dam et al. 2014; Mao et al. 2013; Kameritsch et al. 2012; Robinson et al. 2012; Kawaguchi and Hirano 2013; Simon et al. 2013; Henkin et al. 2007, 2013; Henkin and Velicu 2009; Pechánová et al. 2009; Rukoyatkina et al. 2011; Rodrigo et al. 2006). However, none of these processes were earlier described as related to the liver functions and the metabolism of biologically active compounds. Some data suggest a direct link between guanylate cyclase pathway and gonadotrope cell activation, since there is a link between cGMP and genes involved in reproduction regulations, especially PRKG1 and NOS1. It has been demonstrated that cGMP analogues stimulate GnRH release, while a cGMP-dependent protein kinase (PRKG1) inhibitor conversely blocks nitric oxide-induced GnRH release. Therefore, nitric oxide may regulate the heme-containing signalling enzyme, guanylate cyclase, and thereby elevate the second messenger cGMP and facilitate GnRH secretion. Additionally, an effect of nitric oxide on the heme-containing enzyme should also be taken into account. Cyclooxygenase controls production of prostaglandins, known stimulators of GnRH secretion (Brann and Mahesh 1997).

Taken together, the present study showed that long-term (3 and 14 months) rats feeding with BASE-diet (diet enriches with polyphenolic compounds, β-carotene, probiotics and n-3 and n-6 polyunsaturated fatty acids) can affect liver genes expression. On transcriptomic level, the diet exerted its activity by modulating the expression of genes involved particularly in the gonadotrope cell activation pathway and guanylate cyclase pathway, as well as in mast cell activation, gap junction regulation, melanogenesis and apoptosis. The results indicating the strong influence of BASE-diet on genes involved in the gonadotrope cell activation pathway may suggest the impact of BASE-diet on reproduction system at the transcriptome level. BASE-diet especially strongly affects genes involved in regulation of GnRH which is responsible for the release of FSH and LH. This effect is stronger with the age of animals and the length of diet use. It can suggest a link between the diet, reproductive system function and aging. The reproductive-cell cycle theory assumes that the hormones that regulate reproduction, mainly LH and FSH, act in an antagonistic pleiotrophic manner to control aging through cell cycle signalling (Atwood and Bowen 2011). It means that the improvement of functioning of reproductive system may slow down the rate of senescence, thereby decelerating the rate of aging, and thus the lifespan. It allows us to draw the conclusion that the long-term use of biologically active substances-enriched diet can positively affect the reproductive system, and thus, as a consequence may delay the aging process. However, according to the latest scientific reports, the gonadotrope cell activation pathway plays a part not only in reproduction system, but also in regulation of the aging process. Therefore, the results indicating the strong influence of BASE-diet on genes involved in the gonadotrope cell activation pathway may also suggest the strong impact of BASE-diet on the aging process by the regulation of GnRH-dependent cells. These results indicate that it is highly probable that BASE-diet can modify the signalling pathways which control the aging process by changing the expression of genes involved in gonadotrope cell activation and as a result delay the flow of the aging process.
